# A comparison of ondansetron and lidocaine in reducing injection pain of propofol: a randomized controlled study

**DOI:** 10.1186/s12871-022-01650-4

**Published:** 2022-04-18

**Authors:** Wirat Wasinwong, Sarocha Termthong, Prae Plansangkate, Jutarat Tanasansuttiporn, Riam Kosem, Surewan Chaofan

**Affiliations:** Department of Anesthesiology, Faculty of Medicine, Prince of Songkla University, HatYai, 90110 Songkhla Thailand

**Keywords:** Injection pain, Lidocaine, Ondansetron, Propofol

## Abstract

**Background:**

Direct stimulation of the afferent nerve endings in the venous endothelium is one explanation of propofol injection pain. Previous studies found that ondansetron can also block sodium channels. This effect is similar to that of lidocaine.

**Objective:**

The primary outcome was the efficacy of ondansetron compared to lidocaine and placebo for the reduction of propofol injection pain.

**Method:**

This trial was conducted in 240 patients, American Society of Anesthesiologists classification I-III and aged between 18–65 years old, undergoing elective surgery, and having a 20-gauge intravenous catheter at the hand dorsum. Each group of 80 patients received 8 mg. of ondansetron in the O Group, 40 mg. of lidocaine in the L Group and normal saline in the C Group. The study medications were blindly administered to the patients through a 20-gauge intravenous catheter placed on the hand dorsum, and then 1 min later, the small dose of propofol (50 mg.) was infused via the syringe pump at a rate of 600 ml/hr. for 30 s. Following that, the syringe pump of propofol was temporarily stopped, and the patients were asked to rate their pain at the injection site.

**Result:**

The incidence of pain was lowest in the L group (66.2%) compared with the O (82.5%) and the C groups (85.0%) (*P* < 0.01). The median pain score in the L, O, and C groups were 2 (0–4), 4 (2–5), and 4.5 (2–6), respectively (*P* < 0.01). The incidences of no pain, mild, moderate, and severe pain were also significantly different in the L group (33.8%, 37.5%, 21.2%, and 7.5%, respectively) compared with those in the O group (17.5%, 31.2%, 31.2%, and 20.0%, respectively) and the C groups (15.0%, 22.5%, 40.0%, and 22.5%, respectively) (*P* < 0.01).

**Conclusion:**

Pretreatment with intravenous lidocaine, rather than ondansetron, can reduce the incidence and intensity of propofol-induced pain.

## Background

Propofol is the most commonly used intravenous anesthetic drug**,** which can be administered during induction of general anesthesia or sedation for short procedures. However, injection pain is common and causes patients discomfort during the induction of general anesthesia. The incidence of injection pain has been shown to vary between 28 and 90% [1] and the data from Songklanagarind Hospital found that the incidence of pain was as high as 99%, with a median pain score of 8 (severe pain). Propofol pain was ranked 7^th^of 33 clinical anesthesia dissatisfaction outcomes [2]. The mechanism of propofol injection pain is still unknown, but the theory from many studies found that there were two mechanisms. The first mechanism is the direct irritation of the afferent nerve ending in venous endothelium and the indirect effect from the activation of the Kinin cascade that leads to vasodilation and increases contact between propofol and free nerve endings. In addition, many factors affect pain in propofol injection. These include the size of the intravenous catheter, site of injection, and speed of injection. Various methods have been used to decrease the severity of propofol pain including the propofol administration at the antecubital fossa of the forearm, fast injection of propofol, changing the lipid emulsification form, and pretreatment with lidocaine, opioids, or NSAIDs. The most effective method was the combination of pretreatment with lidocaine and venous occlusion before propofol injection. However, this technique is not clinically practical and not widely used [3].

Ondansetron is a specific serotonin (5-HT3) antagonist. Previous studies found that ondansetron can block sodium channels [4] and opioid receptors [5]. In our practice, ondansetron is routinely administered to prevent postoperative nausea and vomiting. We postulated that pretreatment with ondansetron might decrease the pain on propofol injection. Rahimzadeh P et al. [6] reported that ondansetron had significant impacts on pain reduction after propofol injection in comparison with placebo. Ambesh et al.[7] found that the overall incidence of pain in the control group was 55%, compared with 25% in the ondansetron group and fewer patients in the ondansetron group experienced severe pain (7.5% vs 32.5%). Nevertheless, the results of some studies were inconclusive and most of the studies combined pretreatment medications with venous occlusion, which is not practical.

In this study, we compared the efficacy of 8 mg. ondansetron to 40 mg. lidocaine and a placebo for the reduction of propofol injection pain as the primary outcome. The secondary objective was to compare the incidences of postoperative nausea and vomiting in each group.

## Materials and method

This double-blinded randomized controlled trial was conducted after approval by the Ethics Committee of the Faculty of Medicine, Prince of Songkla University (REC.62–107-8–1) in accordance with the Declaration of Helsinki(1964). It was prospectively registered in the Thai Clinical Trial Registry (www.clinicaltrails.in.th) on July 20, 2019 (TCTR20190720001,20/07/2019). After all of the patients were informed about the study and signed the informed consents, we recruited 240 patients of both genders, American Society Anesthesiologists physical (ASA) status I–III and aged between 18–65 years old, undergoing elective surgeries under general anesthesia in Songklanagarind Hospital, Thailand between August 2019 to November 2020. All of them were inserted with the intravenous catheter number 20-gauge at the hand dorsum prior to surgery.

Exclusion criteria included the patients who weighed < 50 kg.; had difficulty in communicating; were allergic to ondansetron, lidocaine, or propofol; did not receive propofol for the induction, used apomorphine, dronedarone, monoamine oxidase inhibitors and serotonergic drugs; had cardiac arrhythmias especially prolonged QT syndrome, had second and third-degree atrioventricular block; had chronic pain, did not have the intravenous catheter number 20-gauge on the hand dorsum; and received the rapid sequence intubation technique.

All patients were randomly allocated into 3 groups by stratified sampling with the computer program using the method of randomly permuted block. Each group of 80 patients received either 8 mg. of ondansetron (Group O), 40 mg. of lidocaine (Group L) or 0.9% sodium chloride solution (Group C). The random lists were concealed in the envelopes. The random code number was notified for the preparation of total volume of 4 mL. of colorless medications in the 5 mL.-syringe on the morning of the day of surgery by the anesthetist who was not involved in either the administration of study medication or the assessment of the patients. No premedication was administered and the current analgesic drugs were discontinued. A 20-gauge intravenous catheter was inserted into the superficial vein on the hand dorsum and the patients received intravenous fluid infusion on the morning of the day of surgery.

At the operating room, demographic data was recorded by the nurse anesthetists. The leaked, improper placement or dislodgment of the intravenous catheter was checked before the start of induction. All patients were pre-oxygenated with 100% oxygen which was delivered through a facial mask. The study medications were given to the patients through a 20-gauge intravenous catheter placed on the hand dorsum, then 1 min. later, the small dose of propofol (50 mg.) was infused via the syringe pump at a rate of 600 ml/hr. for 30 s. Following that, the syringe pump of propofol was temporarily stopped, and the patients were asked to rate his/her pain at the injection site using a verbal numerical rating score (VNRS) in which score 0 is no pain and score 10 is the worst pain. The residual dosage of propofol was then injected, followed by opioids and neuromuscular blocking agents. Systolic and diastolic blood pressures, heart rates, oxygen saturation, electrocardiogram were monitored and recorded during induction and after intubation. Post-operative nausea and vomiting (PONV) was evaluated at the post anesthetic care unit.

### Sample size calculation

The sample size was calculated with the two-independent proportions formula (two-tailed test) from the propofol pain injection incidence in Songklanagarind Hospital [8]. To reduce the proportion of propofol pain from the study medication by 30%, forty two patients in each group were needed at a significant level (α error) of 0.01 and power of 0.8. We calculated for a 10*%* drop out*.*The definite number of the population was then 47 patients in each group. Moreover, the sample size calculation to cover secondary outcome (PONV) was also used the same formula with data from a previous study [9]. To reduce the proportion of nausea and vomiting from 0.3 to 0.1, the size of the populations required for this secondary objective was 72 patients in each group at a significant level (α error) of 0.05 and power of 0.8. After the calculation for a 10*%* drop out, the final size of the population was 80 patients in each group.

### Statistical analysis

Statistical analysis was performed using Rstudio 1.3.1056. Continuous variables were presented as mean and standard deviation (SD) or median and interquartile range (IQR). Categorical variables were presented as the number of patients and percentages. Continuous variables were analyzed by ANOVA F- test, Kruskal–Wallis test or pairwise Wilcoxon test. Categorical variables were analyzed by Fisher's exact test or Chi-square test. P-value less than 0.01 was considered as statistical significance. The power of this study was 0.8.

## Results

**Fig. 1:**
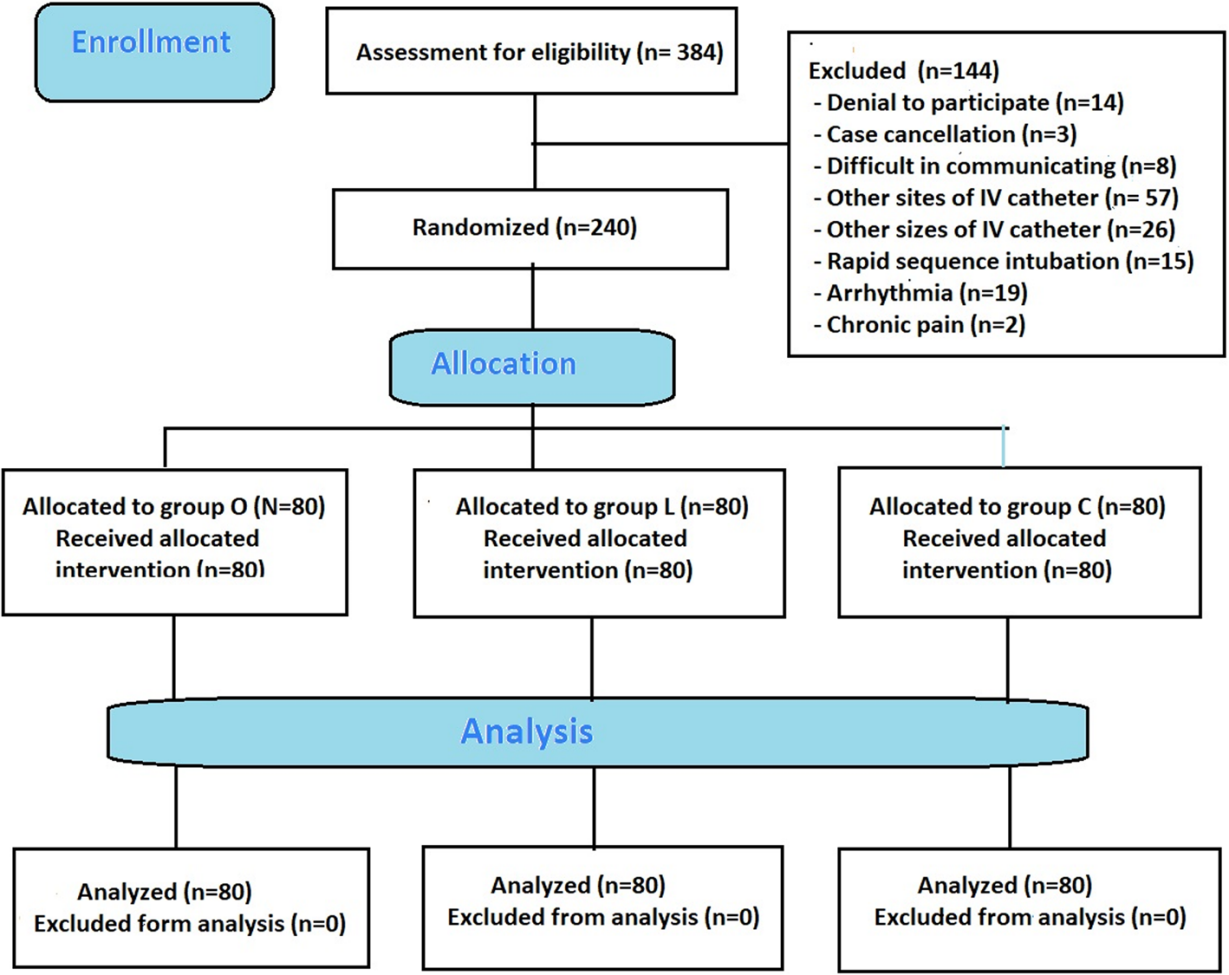
CONSORT diagram of patient recruitment

Three hundred and eighty-four patients were assessed for eligibility from August 2019 to May 2020. One hundred and forty-four patients were excluded and two hundred and forty patients were randomized and then allocated into each group. (Fig. 1) All subjects in each group were analyzed completely. There were no significant differences between the groups regarding gender, age, weight, height, body mass index (BMI), American Society of Anesthesiologist (ASA) physical classification, pulse rate, and blood pressure at baseline, before induction and 1 min after intubation. (Table 1).

**Table 1:** Patients demographic data

	**Group O (*n* = 80)**	**Group L (*n* = 80)**	**Group C (*n* = 80)**	***P*-value**
**Age (years); median(IQR)**	52 (42.8–57.0)	49.5 (38.8–57.0)	47.5 (32.0–58.0)	0.199
**Gender**				
Male; n (%)	22 (27.5)	24 (30.0)	22 (27.5)	0.921
Female; n (%)	58 (72.5)	56 (70.0)	58 (72.5)	
Weight (kg.); median (IQR)	63.5 (56.8–70.0)	65.5 (60.0–71.2)	66 (57.8–74.0)	0.255
Height (cm.); mean (SD)	159.3 (± 8.7)	160.9 (± 7)	160.8 (± 8.9)	0.406
BMI (kg/m^2^); median (IQR)	25.1 (22.8–28.2)	26.2 (23.3–28.5)	24.9 (22.7–29.2)	0.618
ASA Classification; n (%)				
ASA class I	10 (12.5)	12 (15.0)	11 (13.8)	
ASA class II	60 (75.0)	56 (70.0)	58 (72.5)	0.973
ASA class III	10 (12.5)	12 (15.0)	11 (13.8)	
**Baseline; median (IQR)**				
Pulse rate (bpm)	72.5 (65.8–84.0)	75.0 (67.0–85.0)	75.0 (65.0–83.5)	0.665
SBP (mmHg.)	138.5 (125.8–158.2)	145.0 (128.0–160.2)	134.5 (120.0–155.0)	0.247
DBP (mmHg.)	80.0 (70.0–88.2)	81.5 (72.0–90.0)	77.0 (70.0–83.2)	0.066
MAP (mmHg.)	96.5 (87.0–110.0)	101.0 (91.8–109.2)	95.0 (85.8–102.2)	0.046
**Before induction; median (IQR)**				
Pulse rate (bpm.)	70.0 (62.0–81.2)	70.0 (63.8–80.0)	70.0 (60.0–87.2)	0.878
SBP (mmHg.)	121.0 (105.0–140.0)	117.0 (105.0–131.2)	119.5 (96.0–140.0)	0.378
DBP (mmHg.)	78.0 (60.0–85.0)	70.0 (60.0–81.0)	70.0 (55.0–80.0)	0.295
MAP (mmHg.)	87.5 (74.8–100.2)	82.0 (74.0–95.2)	87.5 (66.0–100.0)	0.472
**After intubation; median (IQR)**				
Pulse rate (bpm.)	76.5 (67.8–85.0)	83.0 (70.0–95.0)	75.0 (67.8–88.2)	0.027
SBP (mmHg.)	136.0 (123.8–160.8)	140.0 (123.8–168.8)	130.5 (112.5–155.2)	0.071
DBP (mmHg.)	80.0 (73.5–95.0)	84.0 (67.8–101.2)	78.0 (64.8–90.0)	0.066
MAP (mmHg.)	98.0 (87.5–113.2)	102.5 (85.0–122.0)	92.5 (78.8–107.2)	0.012

**Fig. 2:**
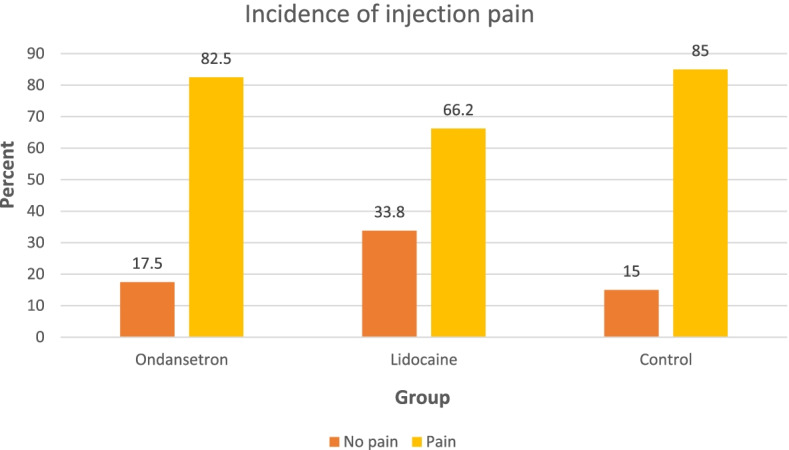
The incidences of propofol injection pain in patients receiving Ondansetron, Lidocaine, or Saline for reducing pain from propofol. Data are proportion of patients with pain (yellow bar) and no pain (orange bar)

**Fig. 3:**
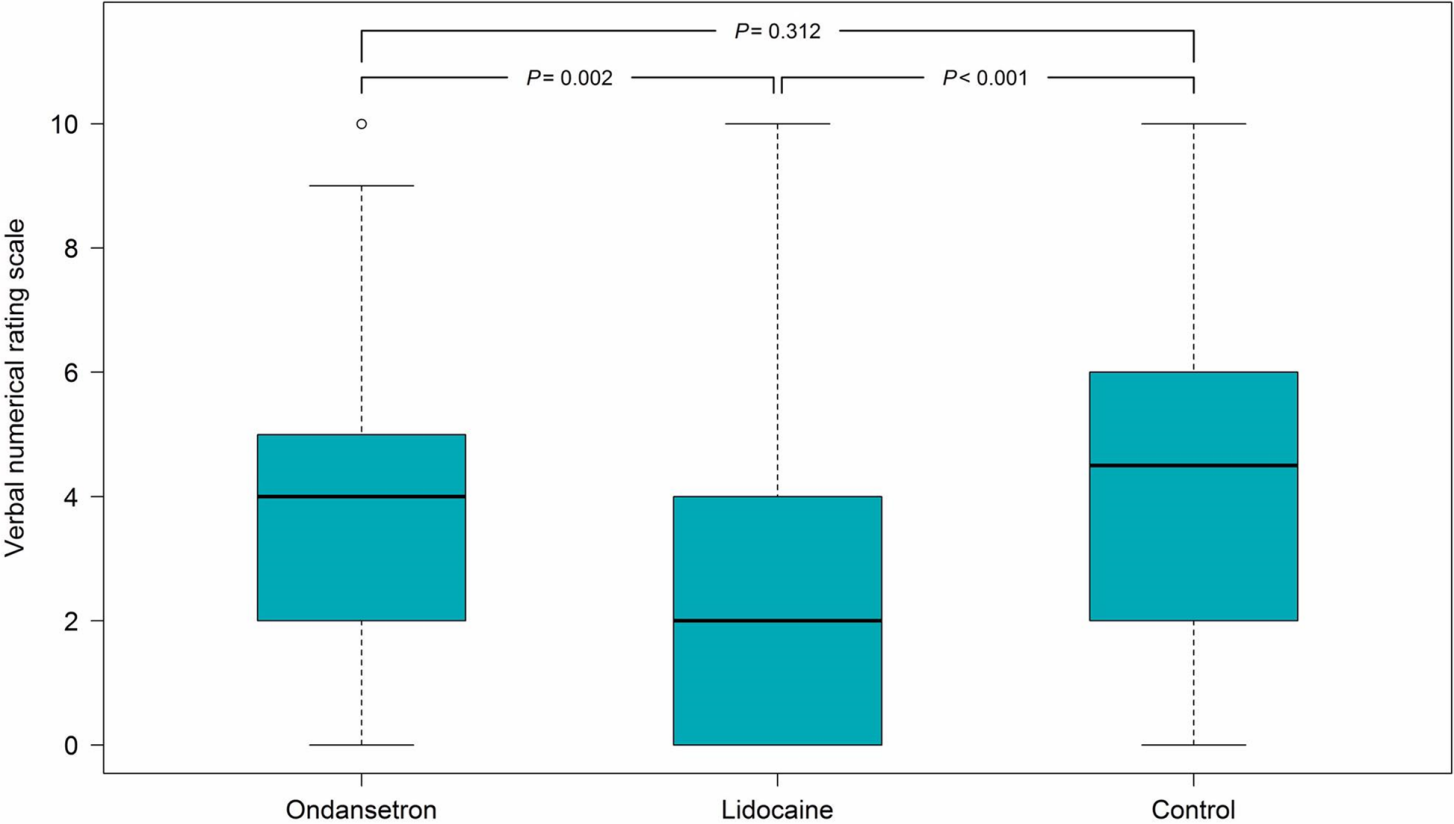
Pain score measured by the verbal numerical rating scale in patients receiving Ondansetron, Lidocaine, or Saline for reducing pain from propofol. Data are median with error bars showing interquartile range

The incidence of propofol injection pain was significantly lower in the L group (66.2%) compared with the O group (82.5%) (*P* = 0.03) and the C groups (85.0%) (*P* = 0.01). (Fig. 2) The incidences of no pain, mild (VNRS of 1–3), moderate (VNRS of 4–6), and severe pain (VNRS of 7–10) were also significantly different in the L group (33.8%, 37.5%, 21.2%, and 7.5%, respectively) compared with those in the O group (17.5%, 31.2%, 31.2%, and 20.0%, respectively) (*P* = 0.01) and in the C groups (15.0%, 22.5, 40.0%, and 22.5%, respectively) (*P* < 0.01). (Table 2) The median pain score in the L, O, and C groups were 2 (0–4), 4 (2–5), and 4.5 (2–6), respectively (*P* < 0.01) (Fig. 3).

**Table 2:** The severity of the propofol injection pain in each group

Pain severity, *n* (%)	Group O (*n* = 80)	Group L (*n* = 80)	Group C (*n* = 80)
No pain	14 (17.5)	27 (33.8)	12 (15.0)
Mild	25 (31.2)	30 (37.5)	18 (22.5)
Moderate	25 (31.2)	17 (21.2)	32 (40.0)
Severe	16 (20.0)	6 (7.5)	18 (22.5)

The *p*-value was 0.01 between group L and O, < 0.01 between group L and C, and 0.52 between group O and C

There were no significant differences between the incidences of postoperative nausea and vomiting among the three groups and no significant complications were reported in all patients.

## Discussion

In this study, we found that pretreatment with the dose of 8 mg. of ondansetron did not reduce the incidence and intensity of propofol injection pain compared to pretreatment with lidocaine with the dose of 40 mg. Compared to the normal saline, it did absolutely not support the analgesic effect of pretreatment with intravenous ondansetron. The result of this negative analgesic effect of ondansetron might be from the many different proposed mechanisms of propofol injection pain.

Propofol causes pain by direct irritation of afferent nerve endings in venous endothelium and the indirect effect from the activation of the Kinin cascade. The activation of the Kinin cascade causes the release of prostaglandin E2 (PGE2), which produces local vasodilatation and increased vascular permeability. In consequence, there is increased contact between propofol and free nerve endings [10]. A meta-analysis conducted by Pei and colleagues [11] showed that ondansetron which is a distinctive 5-HT3 antagonist, can effectively prevent propofol injection pain when combined with occlusion technique, and the effect is similar to that of magnesium sulfate and lidocaine. Ambesh et al. [7] reported that the overall incidence of propofol injection pain in the control group was 55%, compared with 25% in the 4 mg ondansetron combined with venous occlusion technique. Alipour et al. [12], also studied the venous occlusion technique combined with pretreatment of lidocaine or ondansetron. Only one person in the group receiving saline solution (1.7%) had no complaints about propofol injection pain. In contrast, the number of patients without pain was 39 patients in the lidocaine group (69.64%) and 22 patients in ondansetron group (39.28%). The efficacy of pain relief in both ondansetron and lidocaine groups from our study was inferior to these previous studies [7, 11, 12]. The explanation may be due to the venous occlusion technique, which was not applied in our study. The venous occlusion technique was usually performed with a rubber tourniquet on the forearm. The pressure of the tourniquet was between 50 and 70 mmHg, and it was applied for 30 to 120 s. With this combination technique, the number needed to treat (NNT) to prevent propofol pain with 40 mg. of lidocaine compared with 40 mg. of lidocaine only (without venous occlusion technique) was 4.3 to 1.8 [13]. The activation of the nerve fibers responsible for pain transmission resulting from the direct irritation effect of propofol on the inner wall of the blood vessels may be the primary or main mechanism of the injection pain. In addition, the principal mechanism of action of lidocaine as the local anesthetic agent is through the blockade of voltage-gated sodium channels leading to the blockade of action potential propagation that can prevent direct irritation of afferent nerve endings from propofol injection. Therefore, the direct analgesic effect of lidocaine was more effective when there was a long enough duration for the drug to take action during the venous stasis from tourniquet occlusion [14]. Previous studies in animal models reported the ability of ondansetron to block sodium channels and serotonin (5-HT3) receptors [4, 5]. Our hypothesis was that the less analgesic properties of ondansetron by sodium channel block were probably not the principal action of ondansetron. In addition, local anesthetics contain hydrophilic and hydrophobic structures separated by an intermediate amide or ester linkage, a structure that ondansetron does not have. Therefore, ondansetron may have less efficacy even after increasing the dose to 8 mg. in our study.

The factors that are associated with propofol injection pain include the size of the intravenous catheter, site of injection, speed of injection, and lipid solvent of propofol [15]. These confounders were controlled in this study, all patients received the emulsion of 1% propofol in a mixture of medium-chain and long-chain triglycerides as an available preparation in our institution. This study had a lower incidence of propofol pain, compared to the previous study in our institution (85% vs 99%). The possible assumption was the different doses of propofol the patients received before pain assessment. In our study, the propofol dose was 50 mg. in every patient but it was ¼ of the induction dose, which was varied in each patient in the previous study. This 50 mg. dose of propofol may have a sub-hypnotic effect that affects the patient’s interpretation. A previous study revealed a small discomfort issue caused by propofol injection during gastroscopy [16]. As a result, the further research of propofol injection pain should be focused on the sedative procedures.

There were no significant differences between the incidences of postoperative nausea and vomiting among the three groups. This may be due to the wide variation in the surgical duration. Even the 8 mg. of ondansetron is an adequate dose to prevent postoperative nausea and vomiting. However, the time of administration is also important. The surgical duration in our study commonly lasted 4 h., longer than the effective duration of ondansetron which should be within 4 h. The effective antiemetic property of ondansetron usually occur within 30 min. after administration, so it is, therefore, better to administer ondansetron before the end of the operation. One of the lethal adverse effects of ondansetron is prolonged QT which can produce significant arrhythmias. Nevertheless, there was no patient with prolonged QT or arrhythmia in our study.

The limitation of this study was the administration of a sub-hypnotic dose of propofol before pain assessment. Therefore, a reliable pain assessment can be confusing.

## Conclusion

Pretreatment with 8 mg. of intravenous ondansetron before induction did not significantly reduce the incidence and intensity of propofol-induced pain compared to the pretreatment with 40 mg. of intravenous lidocaine. There was either no advantage of prevention of post-operative nausea and vomiting.

## Data Availability

The datasets used and/or analysed during the current study available from the corresponding author on reasonable request.
